# Merit-Based Motion Planning for Autonomous Vehicles in Urban Scenarios

**DOI:** 10.3390/s21113755

**Published:** 2021-05-28

**Authors:** Juan Medina-Lee, Antonio Artuñedo, Jorge Godoy, Jorge Villagra

**Affiliations:** Autopia Program, Centre for Automation and Robotics, CSIC-UPM, Ctra. M300 Campo Real, Km 0.200, Arganda del Rey, 28500 Madrid, Spain; juan.medina@csic.es (J.M.-L.); antonio.artunedo@csic.es (A.A.); jorge.godoy@csic.es (J.G.)

**Keywords:** autonomous driving, motion planning, trajectory generation, speed profile, merit function

## Abstract

Safe and adaptable motion planning for autonomous vehicles remains an open problem in urban environments, where the variability of situations and behaviors may become intractable using rule-based approaches. This work proposes a use-case-independent motion planning algorithm that generates a set of possible trajectories and selects the best of them according to a merit function that combines longitudinal comfort, lateral comfort, safety and utility criteria. The system was tested in urban scenarios on simulated and real environments, and the results show that different driving styles can be achieved according to the priorities set in the merit function, always meeting safety and comfort parameters imposed by design.

## 1. Introduction

Automated Driving Functions (ADF) are progressing at a vertiginous pace. There are already commercial solutions for levels of driving automation 2 to 4 (defined by the Society for Automotive Engineers (SAE) J3016 standard [1]), which are valid in specific Operational Design Domains (ODD), but there are still open problems for a safe navigation in urban environments. In these contexts, decision-making is significantly challenging, as the artificial system must properly interact with a diversity of traffic participants and consider sensors limitations under very different driving situations.

Traditional decision-making methods are often based on predefined rules and implemented as hand-crafted state machines (e.g., [2]). Other classical methods handle the decision-making process as a motion planning problem [3]. More recently, end-to-end solutions (e.g., [4]), enabled by deep and imitation learning, are also achieving impressive performance. Although all these strategies may be successful in many cases, one drawback is that they are designed for specific ODDs and sometimes also produce inexplicable behaviors, which makes it hard to scale them to the complexity of real-world urban driving.

One of the main limitations for most of the state-of-the-art motion planning solutions is their difficulty to provide fallback mechanisms in case of taking a wrong decision, either because of algorithm design limitations or due to sensors/actuators inaccuracy or failure. To overcome this situation, some recent works [5], inspired by high-level cognition mechanisms, propose, instead of looking for a unique and optimal planning/acting solution, to simultaneously map as many reachable states as possible. In this setting, a spatio-temporal picture of the state’s reachability in terms of multiple criteria can be drawn at each planning cycle, paving the path towards a fail-operational strategy. Indeed, with this representation, multiple action areas with similar levels of safety could be identified, allowing one to choose different levels of comfort and utility following eventual sensor uncertainties or actuators misbehavior.

This work proposes following that paradigm, initially designed to model sensory-motor primitives, and applying it to the motion planning problem. To that end, an adaptation of an existing road-oriented path sampling strategy ([6,7]) is proposed here, so that simple primitives can be quickly computed and evaluated in terms of a wide variety of performance indicators. As a result, a framework for behavior generalization would be available in any driving context, allowing not only multiple fallback strategies but also an easy-to-implement and easy-to-interpret mechanism for adaptation to different driving styles.

The main contributions of this work with respect to the existing motion planning literature can be summarized in the four following points:

- Contrarily to the majority of current approaches, it does not seek to obtain a unique (quasi-)optimal solution but a good representation of reachable states. The planning strategy relies on a two-step procedure: (i) a systematic generation of planning-oriented reachability maps, thus allowing it to model all the available trajectories to be followed, and (ii) a multi-criteria evaluation and selection of candidates.
- The proposed algorithm is use-case-independent, as it can consider any driving situation in which drivable corridors are available.
- The multiple trajectory performance indicators computed per motion candidate allow driving style personalization in terms of safety, longitudinal and lateral comfort and utility and could be eventually exploited for fail-operational mechanisms.
- The proposed mechanism is validated in urban-like scenarios, using both a realistic state-of-the-art simulator and an automated vehicle. Note that although the traffic involved in the driving scenes has been limited for the sake of clarity, the framework is fully scalable to much more crowded scenarios.

The outline of the paper is as follows: Section 2 presents an overview of the motion planning architecture. Section 3 describes the generation of the trajectory candidates. Section 4 explains the evaluation method to select the best trajectory among possible candidates. The experimental results are shown in Section 5. Finally, Section 6 presents the concluding remarks.

### Related Work

Motion planning is a key technology of autonomous vehicles that aims to solve the problem of computing a sequence of feasible states for the vehicle to maneuver among obstacles from an initial state toward a desired terminal state, considering the vehicle and actuators restrictions. Despite the extensive research in this field, it still represents a relevant challenge because of unavoidable uncertainties in the operating scenario and computational capability limitations of the ADF.

According to [8], most of these existing decision-systems can be categorized into two major paradigms: mediated perception approaches and behavior reflex approaches. In the former group, several interconnected sub-systems intervene to infer a world model for which the most adapted decision and control are generated. Although this has been the preferred strategy in the automotive industry, extremely concerned with the predictability of safety-critical systems, it may have some limitations when fail-operational behavior is at stake. In the latter group, a direct mapping from the sensory input to a driving action is computed, often supported by different kinds of cognition-inspired mechanisms (e.g., neural networks [9] or reinforcement learning [10]). Although very interesting results can be obtained with this latter approach, the underlying level of abstraction may fail to capture the complexity of a scene, focusing the learning efforts in a wrong direction and, worse, leading to a deficient interpretability of the decisions made by system. To cope with the aforementioned problems, there might be an intermediate approach that is able to provide a safe-by-design representation that directly predicts the affordance of all the available driving actions, allowing a wide variety of fallback mechanisms and a potential oriented learning mechanism.

To address the challenge of learning more efficiently, some authors (e.g., [5]) propose an architecture that covers the complete perception-to-action loop in a biologically plausible model that separates (i) parallel priming on many potential actions or plans (creating some sort of reachability map) and (ii) subsequent adaptive selection, according to different kinds of bias (which could be expressed in terms of mathematical criteria or merit functions).

Inspired by this approach, some relevant works on the motion planning literature could be reoriented to produce not only the most suitable path and speed profile for a given context, but the set of all of them that generate reachable states for the vehicle.

The existing literature on motion planning for autonomous driving can be grouped depending on whether they use sampling or optimization techniques. The latter group proposes solutions to constrained spatio-temporal optimization and receding-horizon control problems (e.g., Model Predictive Control (MPC) [11] or constrained iterative LQR [12]) to compute collision-free trajectories. In the former group of algorithms, a predetermined number of samples is chosen in the sampled space, and the corresponding trajectories are then evaluated with respect to a chosen cost function; they can be classified following the nature of the chosen samples: random (e.g., RRT [13], PRM [14], MPPI [15]) or deterministic (e.g., lattice planners [16], primitives-based [17]). Particularly noteworthy of this category is the focused motion sampling (e.g., [6,18]), where a sampling center that guides the focused trajectory sampling is determined and then random path and velocity candidates are generated and evaluated within this small region.

Although sampling-based strategies are often computationally more efficient, their main associated difficulty is finding the right spatial configuration parameters to obtain a good representation of motion in a given evolution environment [14]. In addition to that, many of these motion planning strategies explicitly include a large set of rules such that the planned trajectories are compliant with the driving situation. These rule-based approaches (e.g., [19]) lack the ability to generalize to unknown situations and to deal with uncertainties. Moreover, under specific circumstances, they need to be relaxed or even violated. If traffic rules are encoded in a merit function, sampling-based motion planning methods can be employed to find the set of authorized and inhibited trajectories, allowing thus parallel behaviors that, properly used, may be exploited in fail-operational decision-making systems.

As a result, the existence of desirable properties in terms of comfort and safety need to be compliant with context-aware utility and translated into appropriate metrics in the state space [20]. Unique proper metrics are often difficult to be defined as the involved costs have coupled and hard-to-model effects. The work from [21] proposes a solution in this direction, where (i) an optimal path is selected from a finite set of path candidates including multi-faceted performance indicators, and (ii) appropriate vehicle acceleration and speed are then generated. However, the article does not give details on the way intermediate waypoints should be generated in a generic environment, limiting its operational scope. In addition to that, the resulting planned speed is not based on a dynamic interaction-aware longitudinal model, which may lead to a suboptimal spatio-temporal exploration. This paper aims at obtaining answers to the aforementioned research questions taking into consideration the current identified limitations.

## 2. Motion Planning Architecture

**Figure 1:**
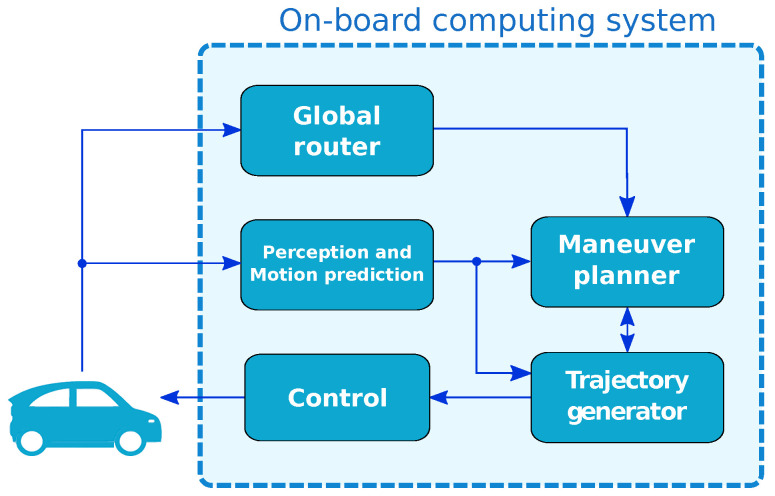
Block diagram of motion planning architecture.

The motion planning architecture proposed in this work is displayed in Figure 1. The system will use a destination point and information of the on-board sensors to generate a proper throttle, braking and steering wheel commands to control the ego-vehicle autonomously.

The perception and motion prediction module uses exteroceptive sensors to estimate the status of traffic agents present on the scene and an occupancy grid $\left(G_{occ}\right)$ of the ego-vehicle surroundings [22]. It also processes GPS and proprioceptive sensors to generate a reliable state estimation (ev) of the ego-vehicle. Finally, this module computes the motion predictions of the vehicles present in the scene $\left(G_{pr}\right)$ by taking into account the interaction between agents. The generation of these motion predictions is described in detail in [23,24].

The maneuver planner module obtains the navigation corridors (ζ) of the ego-vehicle based on its current position on a *lanelet2* map [25]. Each navigation corridor represents a reachable lane for the ego-vehicle in a limited time horizon. Next, the maneuver planner selects the best available corridor (c) using the lane-changing model presented in [26], which allows to evaluate mandatory and discretionary lane-change considerations. This approach of detaching the corridor selection from the trajectory generation allows the motion planner to perform strategic maneuvers like overtaking, getting to the right-most lane before a roundabout, if necessary, or selecting the less occupied lane on a highway. A lane-invasion grid ($G_{inv}$) is calculated from (ζ) in order to evaluate one of the safety indicators of the trajectory candidates computed in the subsequent trajectory generator.

The trajectory generator module creates a valid set of trajectories (Γ) and selects the best of them according to a merit function. Each trajectory consists on a path and a speed profile. The candidates’ paths are created using quintic Bézier curves, which were selected after a thorough comparison [27]. A set of waypoints (ωp), obtained from the centerlines of ζ, are used as ending points for the candidates. The trajectory generator module is the main focus of this paper, and the complete process is described in Section 3 and Section 4.

The global router receives the destination point and calculates the complete route from the current position of the ego-vehicle to the destination. This route is a multi-lane set of lanelets that the maneuver planner will use to create the possible navigation corridors. Finally, the control module is in charge of calculating the proper throttle, braking and steering wheel commands to follow the best trajectory from the trajectory generator module. This module has been tested in an automated vehicle with good results in [28].

The Algorithm 1 shows the steps of the proposed motion planning process. It receives the destination point (d) and the data (ψ) from the on-board sensors as inputs. The output of the algorithm is a suitable trajectory $\left(\Gamma_{best}\right)$ for the vehicle control module to follow. A new iteration of the motion planing algorithm starts over as soon as the previous iteration is finished, so the current trajectory is constantly being updated, allowing the system to react properly in dynamic environments. The algorithm keeps running on a loop until the destination point is reached.

**Algorithm 1:** Motion planning algorithm

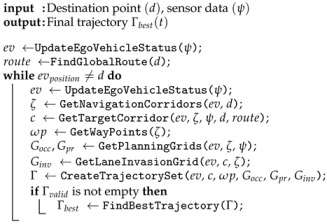



## 3. Generation of Candidates

This section describes the trajectory generation process as well as the preparatory steps required for this purpose.

### 3.1. Navigation Corridors Computation

The first step is to obtain a set of possible navigation corridors. The length of each corridor is equal to the maximum distance that the ego-vehicle can reach on a fixed-time window given its current speed and assuming maximum acceleration. *Lanelet2* maps format [25] was chosen since it provides two layers of information: physical and topological. The physical layer contains geographic information such as borderlines, centerlines or location of elements, while the topological one contains the relation between road elements in a graph network.

**Figure 2:**
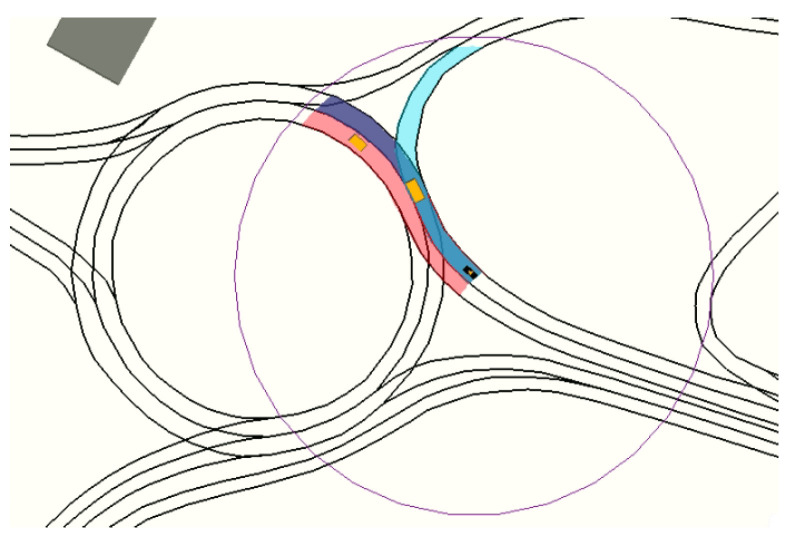
Navigation corridors for the black vehicle on a roundabout scenario.

In order to compute ζ, the lanelet(s) where the ego-vehicle is located is (are) found by evaluating the position and orientation in the physical layer of the map. Next, a graph search on the topological layer of the map is performed to create a lanelet sequence for each corridor. Figure 2 shows the navigation corridors for the ego-vehicle (black vehicle) in a roundabout scenario using a time-window of 5 s and a maximum possible acceleration of 3 m/s${}^{2}$.

In this case, there are three possible corridors: two of them (blue and red) stay inside the roundabout, and a third one (cyan) takes the first exit to the right. The purple circle around the ego-vehicle represents the planning horizon for the corridors in this particular context.

Once ζ is created, the next task is to select the best corridor c∈ζ. This calculation is performed using Toledo’s lane-changing model [26], which assigns a utility level and evaluates the gap acceptance for each corridor. The chosen corridor is the one with the greatest utility that has an acceptable gap.

Finally, a set of waypoints ωp is computed from the centerlines of ζ. These waypoints will be used in the candidate paths generation.

### 3.2. Planning Grids

Three different grids are involved in the evaluation of the trajectory candidates: an occupancy grid $\left(G_{occ}\right)$, a lane-invasion grid $\left(G_{inv}\right)$ and a motion-prediction grid $\left(G_{pr}\right)$.

**Figure 3:**
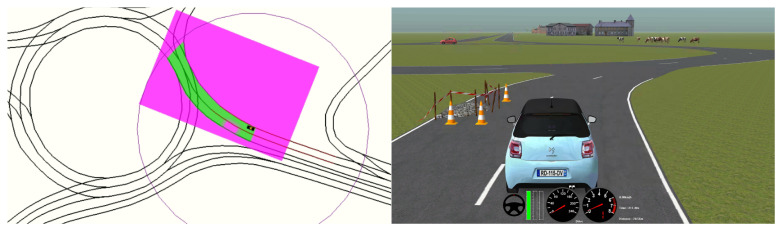
Occupancy grid for planning.

$G_{occ}$ contains information about the static obstacles present on the driving scene and information of the navigation space obtained from ζ. Figure 3 shows an example of $G_{occ}$ in a roundabout scenario with static obstacles on the left lane. Green cells are considered free ($P_{occ}=0$), while magenta cells are occupied ($P_{occ}=1$). The cells around the static obstacles have intermediate values that are used in the evaluation of the candidates; the closer to the obstacle, the higher the value of the cell. Thus, candidates further from obstacles are preferred. Any candidate that goes through a completely occupied cell is considered as not valid.

**Figure 4:**
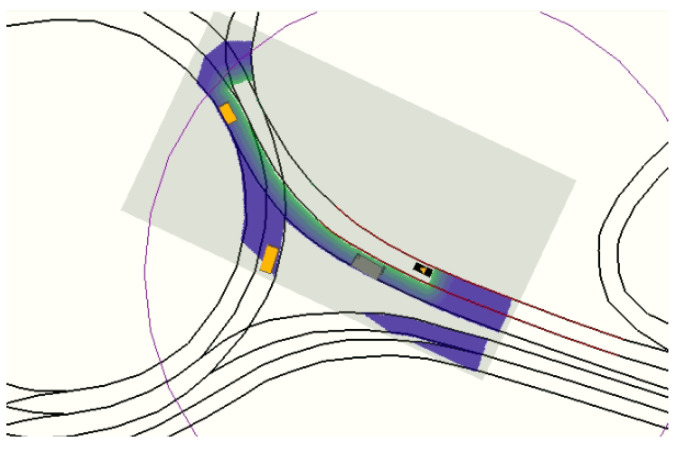
Lane invasion grid in the entrance of a double-lane roundabout.

$G_{inv}$ is a grid used to quantify the candidates’ invasion level into adjacent lanes. The cells inside the ego-lane are set to 0, whereas the value of the outside cells is calculated based on the Euclidean distance to the border of the ego-lane, using the following expression:

(1) $$G_{inv,j}=\frac{min(db_{j},db_{max})}{db_{max}}$$

where $db_{j}$ is the Euclidean distance of the cell *j* to the border of the ego-lane and $db_{max}$ is a design parameter to define the maximum invasion distance into adjacent lanes. In this case $db_{max}$ is set to 3 m, so any cell with $db_{j}>3$ will have a $G_{inv,j}=1$. Figure 4 shows $G_{inv}$ for the same scenario as Figure 3, the cells inside the current lane are plotted in white, while cells inside the road but outside the ego-lane are fading into blue, which represent $G_{inv,j}=1$. The cells outside the road are not computed, and therefore their default value is 0.

**Figure 5:**
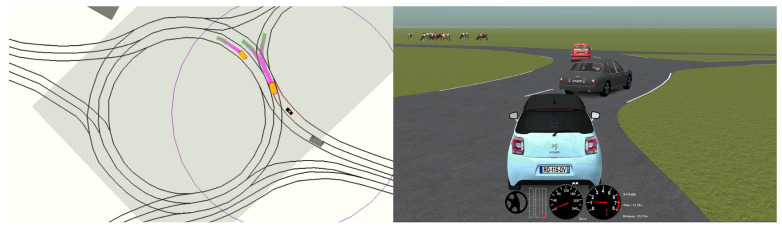
Combined predictions grids for two vehicles in a time horizon of 4 s.

$G_{pr}$ is a set of grids, where each grid contains the estimated positions of the traffic agents within a finite time interval from the current instant $t\in [0,t_{f}]$. Each cell has a binary value: free or occupied by a vehicle in the future. Figure 5 shows the combination of the prediction grids for two vehicles inside a roundabout. Magenta cells represent the predictions at t=0, while green cells represent the predictions at t=4 s.

### 3.3. Path Generation

Each iteration, a new set P of possible paths for the ego-vehicle to follow is created. Each path candidate (ρ∈P) must be consistent with the current trajectory (which is being used as reference by the control module) with the purpose of getting a smooth navigation. This consistency is achieved by maintaining the initial segment of the current path and generating the new paths from a point located at a distance $d_{init}$ ahead of the ego-vehicle. The ending point of ρ is a waypoint ep∈ωp. The location of the starting and ending points of the Bézier curves of the candidates are already determined, and the remaining control points are established using the algorithm described in [18], which allows to explore a large variety of possible paths while maintaining $G^{2}$ curvature continuity as well as imposing orientation in the starting and ending points.

**Figure 6:**
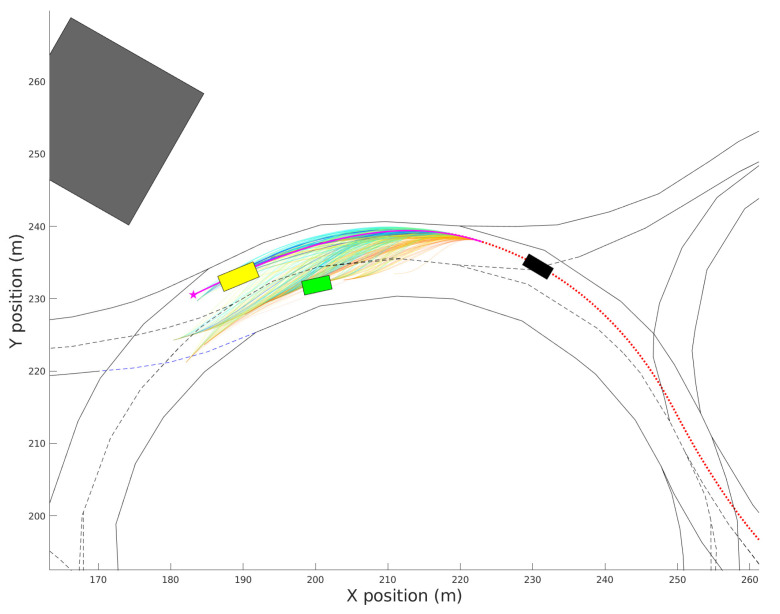
Candidate generation to different navigation corridors.

A validity check is performed before the generation of speed profiles and the merit evaluation of the trajectory candidates, so only valid path candidates are converted into complete trajectories. Each ρ is considered as valid if (i) its maximum curvature is lower than the maximum curvature feasible by the ego-vehicle and (ii) the area occupied by the vehicle while driving along the path does not include any completely occupied cell in $G_{occ}$. Figure 6 shows the valid path candidates for a roundabout scenario with two obstacle vehicles (yellow and green). The ego-vehicle (black) is behind the obstacles, and the candidates are generated from a future position of the current trajectory to the waypoints located in the centerlines of the navigation corridors. The red-dotted line shows the trajectory trail followed by the ego-vehicle until the current iteration. The best trajectory candidate, whose selection process is described in Section 4, is highlighted in magenta.

### 3.4. Speed Profile Generation

The valid paths are converted into trajectories by assigning them a speed profile. The speed profile generation algorithm limits the lateral and longitudinal accelerations to satisfy comfort requirements, and it also uses a dynamic inter-distance model to maintain safe-distance from obstacles.

The first step is to create an obstacle-free speed profile considering, on the one hand, the geometry of the path, and on the other hand, lateral and longitudinal comfort accelerations bounds. Next, a traffic-based speed profile is created taking into account the obstacles present on the scene and using the obstacle-free speed profile as its maximum limit. The traffic-based speed sequence is then assigned to the path to create a trajectory candidate. If a valid trajectory has already been selected in a previous iteration, the speed profile of that trajectory is maintained during $d_{init}$ meters in the new candidates in order to keep a stable reference for the control module.

The future positions of the obstacles are projected into a spatio-temporal representation for each path, with the purpose of including them in the speed profile generation. This projection is performed by evaluating the occupancy polygon of each valid path into the prediction grids set $\left(G_{pr}\right)$ and finding the intersections. The projected occupied space of the obstacles that have intersecting points with the path will be referred to as Possible Collision Points (PCP). The PCPs also contain information about the ID and speed of the obstacles.

**Figure 7:**
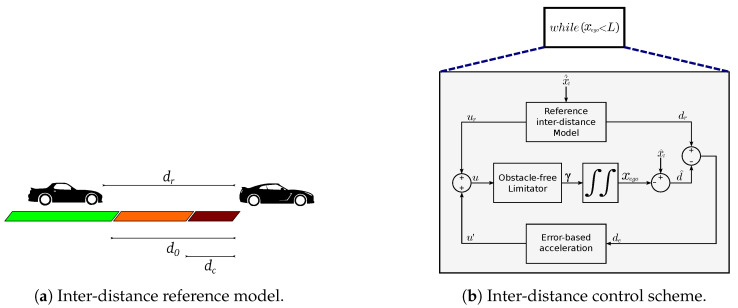
Speed profile generation.

The traffic-based speed profile is created using the inter-distance model proposed in [29]. This reference model creates a virtual vehicle located at a reference distance $d_{r}$ from a leader vehicle and determines the required acceleration for the ego-vehicle to keep such distance (see Figure 7a). The PCPs are used to feed this model with the leader vehicle positions $\left(\hat{x_{l}}\right)$ and speeds $\left(\hat{\dot{x_{l}}}\right)$ so that the ego-vehicle keeps a safe-distance to these points. The inter-distance model only influences the acceleration of the ego-vehicle when the distance to the leader vehicle $\left(\hat{d}\;\right)$ is lower than a safe nominal constant inter-distance $d_{0}$, and the resulting speed-profile will stop the ego-vehicle before reaching the design minimal inter-distance $d_{c}$. An acceleration $u^{\prime }$ that minimizes the tracking error signal $d_{e}$ is put together with the reference-model acceleration and then applied to the ego-vehicle as (γ), while considering the obstacle-free speed-profile along the path. This speed-profile generation process is repeated at each planning step for all the path points (i.e., $x_{ego}$ is lower than the length of the path (L)), as represented in Figure 7b.

The detailed algorithms for the creation of obstacle-free speed profiles, PCPs and traffic-based speed profiles can be found in [30].

In order to increase the diversity of trajectory candidates and provide the candidate-selection module a richer trajectory set (Γ), the acceleration limits $\left(\gamma_{l}\right)$ of each candidate’s speed profile are generated with a bounded random value, as shown below:

(2) $$\gamma_{l,i}=\varphi_{i}*\frac{\gamma_{o,i}}{2}+\frac{\gamma_{o,i}}{2},\;\;\;i=1,2,3,4$$

where $\varphi_{i}\in \left[0,1\right]$ is a random number with a uniform distribution and $\gamma_{o}$ is a vector formed by the four acceleration limits involved in the speed-profile generation, which are presented in Section 5.2.

**Figure 8:**
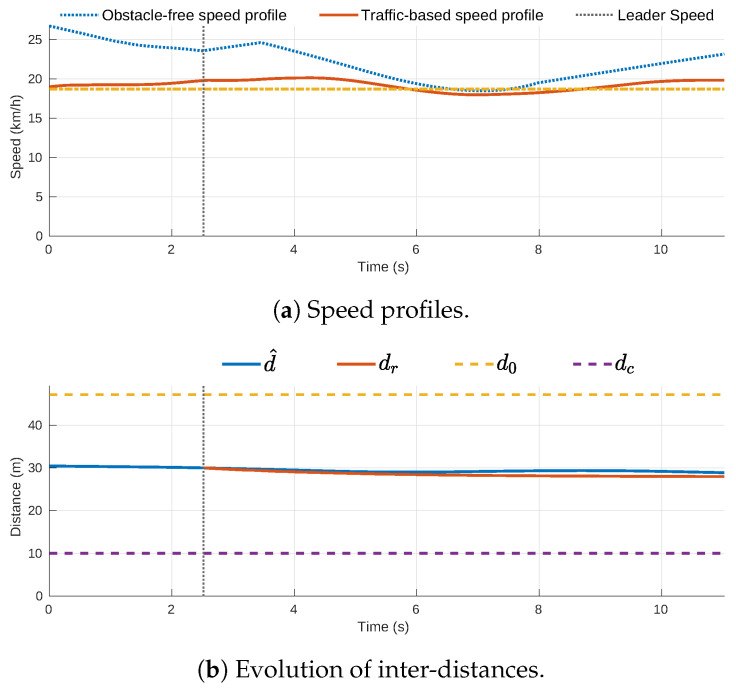
Speed profile generation for the best trajectory candidate in the roundabout traffic scene.

Figure 8a shows the obstacle-free speed profile and the traffic-based speed profile for the best trajectory candidate on the traffic scene of Figure 6, as well as the speed of the leader vehicle. The vertical line in t=2.51 s indicates the ending point of the initial section where the speed profile of the previous trajectory is kept. Figure 8b shows the estimated inter-distance evolution $\left(\hat{d}\right)$, the reference inter distance $\left(d_{r}\right)$, the safe nominal constant inter-distance $d_{0}=47.1$ m and the minimum possible inter-distance between vehicles $d_{c}=10$ m (set by design). The tracking error signal $d_{e}$ increases slightly around t=6 s due to the obstacle-free limitations but is reduced again after t=10 s.

## 4. Evaluation of Candidates

Once the trajectory set (Γ) is created, the motion planner must decide which is the best candidate to handle the current driving scene. This task is performed by comparing the candidates using 4 Decision Variables (DV): longitudinal comfort, lateral comfort, safety and utility. Each DV is obtained from a set of Trajectory Performance Indicators (TPI) that measure different variables of the candidates such as accelerations, jerks, distance to obstacles and lane invasion, among others. The merit of the candidates is quantified by combining the DV using a weighted function that allows the system to select one candidate or another according to the driving profile. For example, if the driving strategy is to drive comfortably, the system will prioritize candidates with lower accelerations and jerks even if that has a negative impact in the cruise speed; if the driving strategy is rather aggressive, then the candidates with a higher utility will be preferred, in spite of reducing safety or comfort.

### 4.1. Trajectory Performance Indicators and Decision Variables

A set of 15 TPI is computed for each trajectory candidate, grouped into four DV, as shown in Table 1. According to [31], comfort in autonomous vehicles is directly related to acceleration (γ) and jerk (ȷ), which explains their central role in comfort DV. The longitudinal comfort variable is obtained by combining the mean and maximum values of the longitudinal acceleration and jerk. The lateral comfort not only combines the lateral acceleration and jerk, but it also includes the smoothness of the path, obtained from the first and second derivative of its curvature, as in [18]. In [32], the collision risk is minimized by increasing the distance to the existing obstacles in the driving scene; in [33], the authors propose different metrics such as time headway or lateral and longitudinal distances to surrounding obstacles, and in [34], the risk metric is directly linked to the lane departure of the vehicle. With this in mind, the safety DV is computed by measuring the distance to static obstacles, the average inter-distance to a leader vehicle (if present) and the lane invasion of the candidates. Most of these TPI are obtained by evaluating the occupancy polygon of the candidate in the planning grids $G_{occ}$ and $G_{inv}$. Finally, the utility is calculated from the average speed of the candidate and the length of its path. In the end, each TPI is normalized using a maximum possible value set by design.

### 4.2. Merit Function

The merit score (m) assigned to each candidate is calculated by combining the four DV with a modified version of the weighted product (WP). This approach was chosen over the weighted sum (WS) because it provides an intrinsic filter (due its multiplication nature) to the candidates that do not perform well in one of the DV [35]. For the sake of illustration, if a candidate has an outstanding longitudinal comfort but keeps a very dangerous inter-distance with the vehicle up front, it should be considered irrelevant. Each DV is first weighted using a non-linear weighting function in order to vary its influence in the final merit of the candidate, and then it is multiplied with the other DV:

(3) $$m_{c}=\sqrt[{4}]{\prod_{j=1}^{4}wf\left(DV_{c,j}\;,\;\omega_{j}\right)}$$

where $DV_{c,j}$ represents one of the four decision variables of a candidate *c* and $\omega_{j}\in \left[0,1\right]$ is the weight of that DV.

This weighting function is designed to satisfy three properties: (i) it must reinforce the difference between the lower and higher values of $DV_{j}$ when $\omega_{j}\to 1$; (ii) it must decrease the difference between the lower and higher values of $DV_{j}$ when $\omega_{j}\to 0$; (iii) values of $DV_{j}$ near 0 must stay near 0 after being weighted. The weighing function is defined to be bounded in the interval [0,1]. In order to meet these properties, the weighing function $wf(DV_{j}\;,\;\omega_{j})$ is defined as a piecewise function which depends on the value of $\omega_{j}$. If the value of $\omega_{j}$ is lower than 0.5, the function is similar in shape to the square root function, and if the value of $\omega_{j}$ is higher or equal than 0.5, the weighting function has an exponential behavior. The formal definition of this function is as follows:

(4) $$wf\left(DV_{j}\;,\;\omega_{j}\right)=\left\{\begin{array}{cc} \frac{1-\omega_{j}^{2\;DV_{j}}}{1-\omega_{j}^{2}} & if\;\;DV_{j}<0.5\\ DV_{j}^{2\;\omega_{j}} & \mathrm{otherwise} \end{array}\right.$$

**Figure 9:**
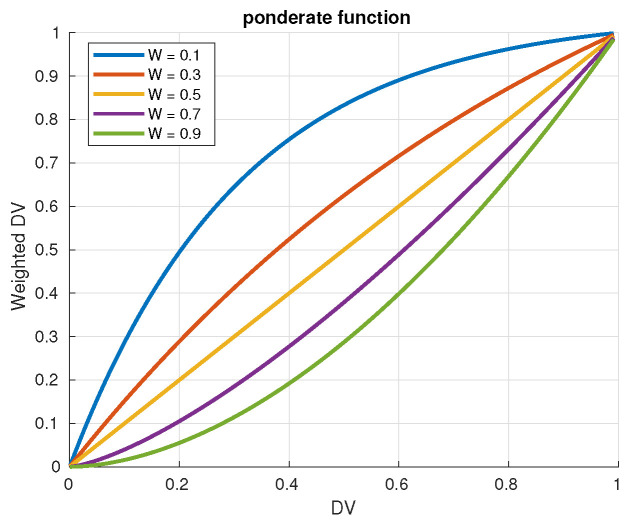
Behavior of non-linear weighting function wf(DV,ω) for different values of ω.

Figure 9 shows how a DV is modified with different values of ω.

**Figure 10:**
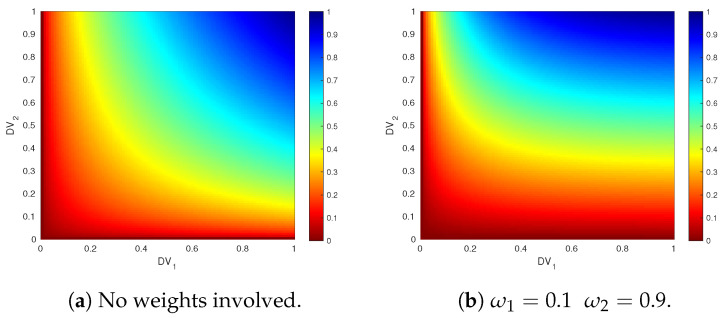
Weighing function for different weight configurations of two parameters.

Figure 10 illustrates the performance of the merit function (Equation (3)) when combining two different DV. Figure 10a shows the merit values after combining the two DV with a non-weighted geometric mean, where it can be seen that both DV equally affect the resulting merit. In Figure 10b, $DV_{1}$ and $DV_{2}$ have weights $\omega_{1}=0.1$ and $\omega_{2}=0.9$, respectively. In this case, the variation of $DV_{1}$ does not influence the merit function as much as the variation of $DV_{2}$, but it gets very close to 0 when values of $DV_{1}$ are near to 0, as expected for property (iii).

Each DV is created from a combination of TPI, as stated in Table 1, using the merit function (3). Accordingly, if a candidate performance is poor for a specific TPI, then the value of the corresponding DV will be low, and the final merit of that candidate will be affected. Since TPI are defined using a lowest-is-better equation and WP needs a greatest-is-better formulation, they are inverted after being normalized, using, for each of the 15 TPI, the following expression:

(5) $$TPI_{k}=1-TPI_{k},\;\;\;k=1,\ldots ,15$$

**Figure 11:**
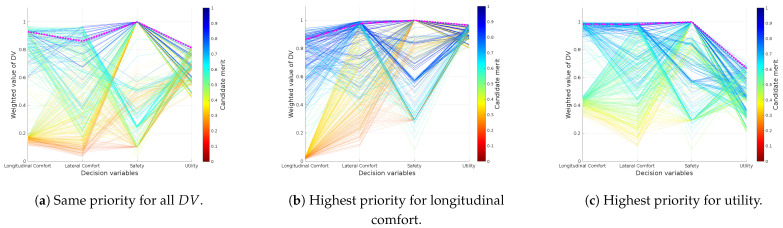
Reachability maps of the trajectory set (Γ) when different weight configurations are applied to *DV*.

**Figure 12:**
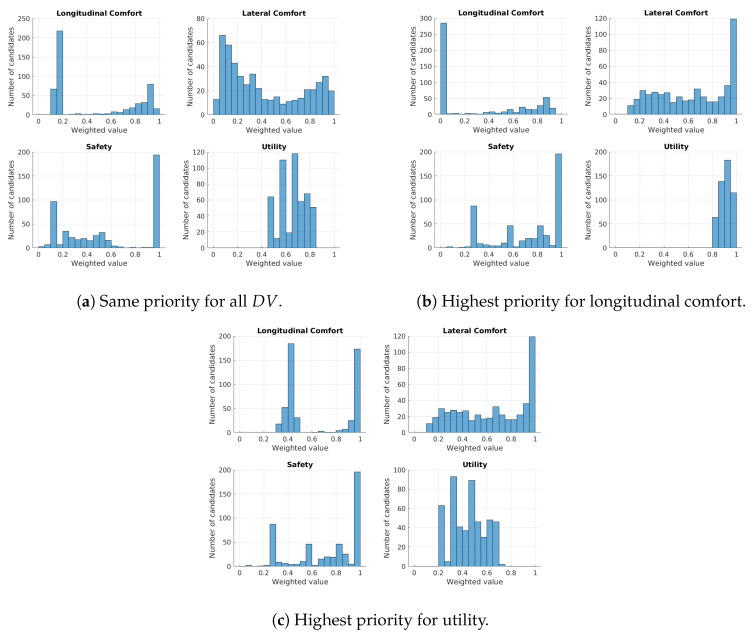
Histogram of the *DV* of Γ when different weight configurations are applied.

In order to show how the weight configuration affects decision making in the autonomous driving process, three different weight configurations (see Table 2) were tested for the driving scenario of Figure 6. Figure 11 shows a reachability map where each candidate is represented as a vector, formed by four decision variables $\left(DV_{j},\;j\in \left[1,\ldots ,4\right]\right)$ and their correspondent weighted value. The final merit assigned to the candidates is plotted using a color-map. Alternatively, Figure 12 shows the distribution of the weighted DV in a histogram representation.

The first configuration, where each $DV_{j}$ has a weight $\omega_{j}=0.5$, is used as baseline (see Figure 11a and Figure 12a to see its $DV_{j}$ distribution). The second configuration establishes the highest weight to the longitudinal comfort and sets the weights of the other DV to 0.1. The effect of this configuration is that the system will be more selective with the candidates according to their longitudinal comfort, and the other DV will not affect the final merit correspondingly. This behavior can be observed in the histogram distribution of the Figure 12b. Note that in the case of the longitudinal comfort, a great number of candidates obtained a value lower than 0.05, while the performance of the other DV was improved compared to the distributions of Figure 12a (the number of candidates in the higher bins of the histograms increased). Figure 11b shows that the overall merit of the candidates was increased and that candidates with poor longitudinal comfort performance tend to have low merits, while candidates with better longitudinal comfort performance have a higher merit. In the third configuration, the highest weight is assigned to utility; as a result, the system raises the bar with this DV, and only the candidates with good utility performance maintain a good value after being weighted. Now the number of candidates tend to be more distributed along the utility histogram, as seen in Figure 12c; besides, the performance of the candidates with regard to the longitudinal comfort increased considerably compared with Figure 12b, and now it does not influence the final merit of candidates as much (there are no red candidates due to the low performance on the longitudinal comfort). In this case, the overall merit of the candidates improved because of the low influence of DV such as safety or longitudinal comfort in this configuration.

**Figure 13:**
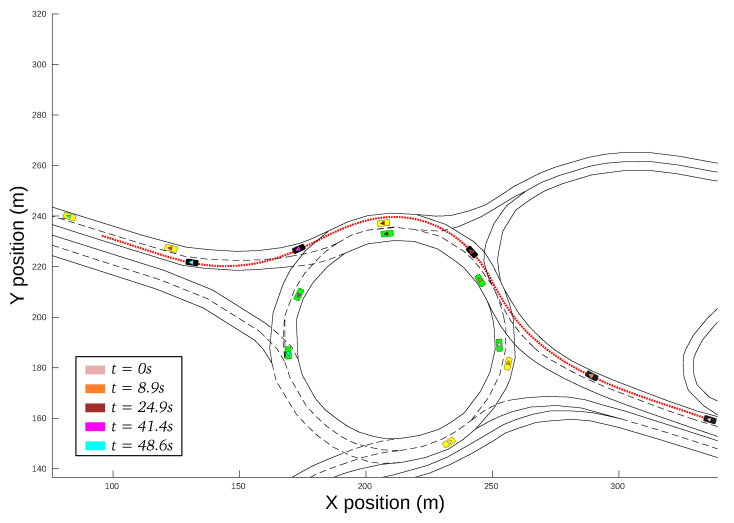
Vehicle evolution during the complete driving scenario.

Figure 13 shows the vehicle’s evolution inside the roundabout during the complete driving scenario. It can be observed that the ego-vehicle keeps a distance with respect to the yellow vehicle inside the roundabout, and it performs a lane-change in the highway once outside the roundabout. The trajectory does not follow the centerline of the ego-lane in order to increase the lateral comfort, but it does not invade the adjacent lane because that would jeopardize safety. This experiment was carried out with a weight configuration wc=[0.5,0.5,0.5,0.5].

## 5. Experimental Results

The performance of the motion planning algorithm was tested both in a simulation environment and in a real vehicle driving on a test track. This section shows the results after performing a number of experiments with different weights configurations and analyzing how the final trajectory and the driving profile is affected by those weights. For the performed experiments, 650 candidates were created at each planning cycle in the Trajectory generation module.

### 5.1. Testing Environments

**Figure 14:**
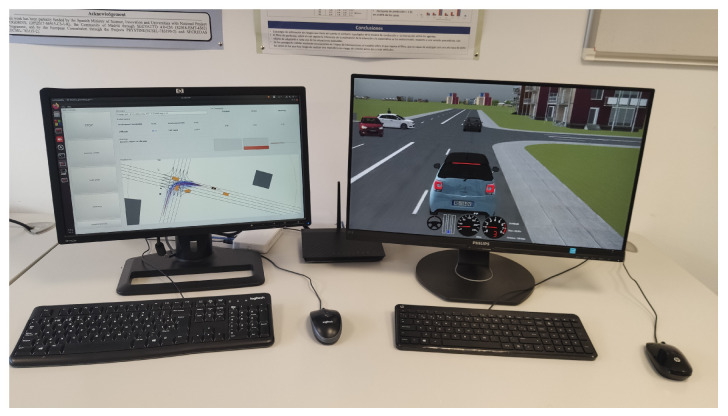
Simulation testing environment.

In the simulation environment, the motion planner was connected in a software-in-the-loop architecture to SCANeR Studio 1.9 simulation software [36]. A middleware was implemented in [37] to allow a real-time interaction with SCANeR Studio by using the same commands applied to control the real vehicle, so the motion planning software was the same for simulation and real environments. Figure 14 shows a setup where the simulation environment reproduces a double-lane intersection (right-side screen) and the motion planner controls the vehicle in real-time on the left-side screen.

**Figure 15:**
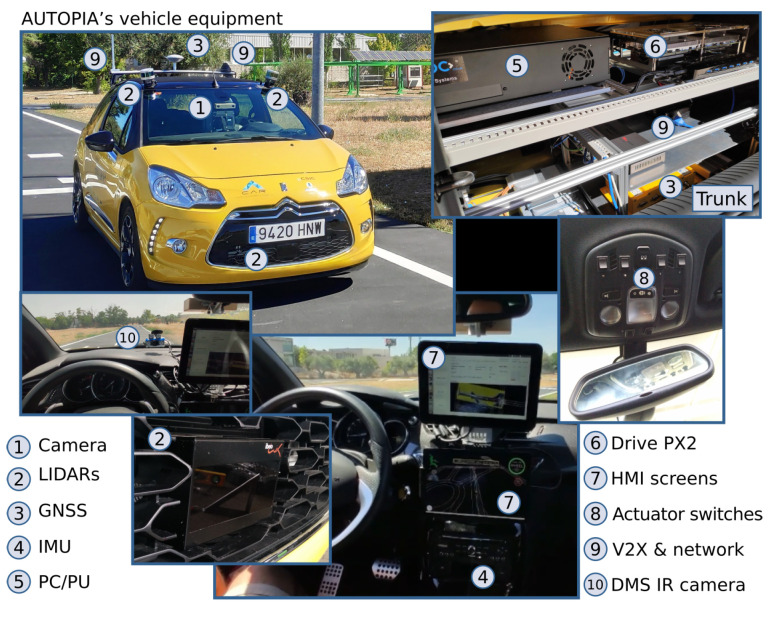
Modules of the autonomous vehicle of AUTOPIA Program.

The automated vehicle on which the algorithm was evaluated is a Citroën DS3 with different exteroceptive and proprioceptive sensors, actuators and high-end computing devices, which allow testing complex algorithms. The main modules installed on the vehicle are listed in Figure 15. The experiments involving the automated vehicle were carried out in a testing track that includes intersections and roundabouts.

### 5.2. Experimental Setup

The methodology used to test the Trajectory generator and the influence of the DV weights in the driving profile was to (i) repeat the same maneuver (defined by a layout, an initial configuration and a final point) using different weight configurations and (ii) to analyze offline the final trajectories using a set of key performance indicators (KPI) to compare the results.

Each scenario was repeated five times. In the first four, a different DV was prioritized by assigning to it the maximum possible weight (1.0), while the weights of the other DV were set to 0.1, reducing thus their influence on the driving behavior without canceling it. In the fifth configuration, all DV had a weight of 0.8, which forces candidates to have good performance in all of DV. Table 3 shows the numeric values of the weight configurations used in the experiments.

The acceleration/deceleration limits used in the speed profiles generation for these experiments are showed in Table 4. The obstacle-free speed profile involves the parameters $\gamma_{comf,lat}$, $\gamma_{comf,acc}$ and $\gamma_{comf,dec}$; while the traffic-based speed profile uses $\gamma_{comf,acc}$ and $\gamma_{safe,dec}$ parameters.

The limits of $\gamma_{comf,lat}$, $\gamma_{comf,acc}$, $\gamma_{comf,dec}$ were selected to comply with the comfort limits proposed in [31], which recommends acceleration values between 1 m/s${}^{2}$ and 1.4 m/s${}^{2}$. The value of $\gamma_{safe,dec}$, obtained empirically, limits the maximum possible braking acceleration and keeps proper inter-distances to guarantee a safe braking.

The KPI used to analyze the experiments are shown in Table 5. They are grouped in four Comparing Categories (CC) that match the decision variables used in the trajectory generator module. The longitudinal comfort, lateral comfort and utility CC are computed by combining two KPI using a weighted arithmetic mean:

(6) $$CC_{j}=\alpha_{1}\;KPI_{1,j}+\alpha_{2}\;KPI_{2,j}\;,\;\;j=1,2,4$$

Weights $\alpha_{1},\alpha_{2}$ are tuned so that both KPI of each $CC_{j}$ have the same influence on the result. This is achieved by using the equation:

(7) $$\left\{\begin{array}{cc} \alpha_{1}=1,\;\alpha_{2}=\frac{\bar{KPI_{1}}}{\bar{KPI_{2}}}, & if\;\;\bar{KPI_{1}}>\bar{KPI_{2}}\\ \alpha_{1}=\frac{\bar{KPI_{2}}}{\bar{KPI_{1}}},\;\alpha_{2}=1 & \mathrm{otherwise} \end{array}\right.$$

where $\bar{KPI_{1}}\;,\;\bar{KPI_{2}}$ are the mean values among the performed experiments.

The longitudinal comfort of the final trajectory was calculated from the average magnitude of the longitudinal acceleration and jerk. Lateral comfort was obtained in the same way, but considering the lateral acceleration and jerk. Safety was determined from the lane invasion along the route; this KPI was calculated by integrating the invasion distances of the vehicle to adjacent lanes $\left(d_{li}\right)$. Lastly, the utility was computed from the positive acceleration and average speed KPI. The positive acceleration is the integral of the positive values in the acceleration profile, the purpose of this KPI being to quantify how aggressive the longitudinal acceleration profile was.

Once the CCs are computed from the KPI, they are normalized using the maximum value among each category and inverted to obtain a higher-is-better format.

#### 5.2.1. Simulation Experiments

**Figure 16:**
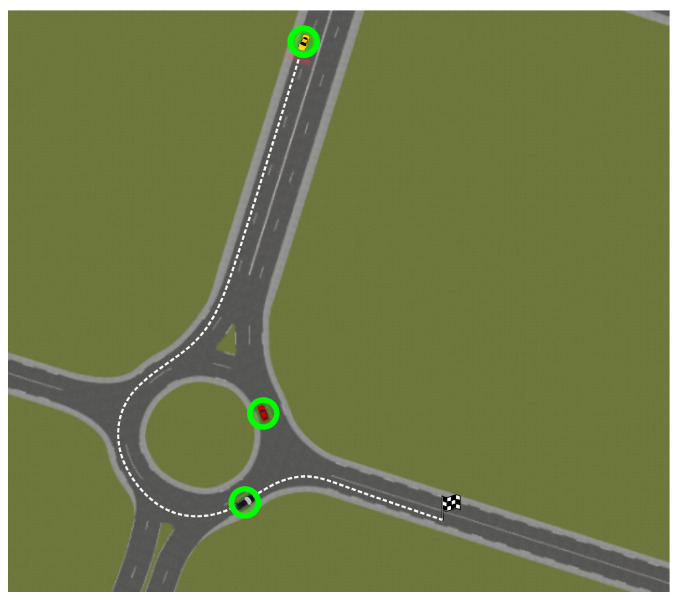
Experimental setup for simulation environment.

The simulation scenario is shown in Figure 16. It consists of a roundabout with two moving vehicles inside. The ego-vehicle will go into the the roundabout, reducing its speed to avoid collision with the obstacles and will take the third exit of the roundabout to reach the destination point. The speeds of the red and black vehicles are 15 and 17 km/h, respectively, both constant throughout the whole scenario.

**Figure 17:**
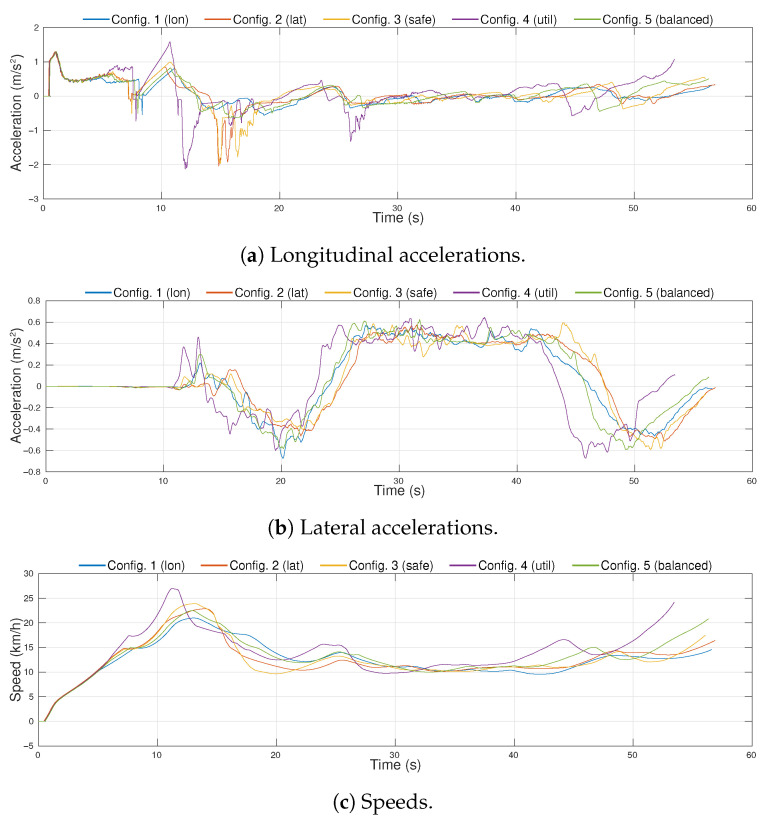
Acceleration and speed profiles for the different configurations executed in the simulation environment.

The resulting accelerations and speed profiles of the ego-vehicle in the five experiments are plotted in Figure 17. From the longitudinal acceleration profiles (Figure 17a), it can be observed that Configuration 1 and Configuration 5 had smoother acceleration and their maximum values were small, while the accelerations used in Configuration 4 were the highest. Regarding lateral accelerations (Figure 17b), even though all profiles had a maximum value of ∼0.6 m/s${}^{2}$, in Configuration 2, the values were steadier, hence reducing the lateral jerk of the trajectory. The speed profiles, plotted in Figure 17c, show that Configuration 4 reaches the highest value and it gets to the destination point around 4 s before the rest of configurations.

**Figure 18:**
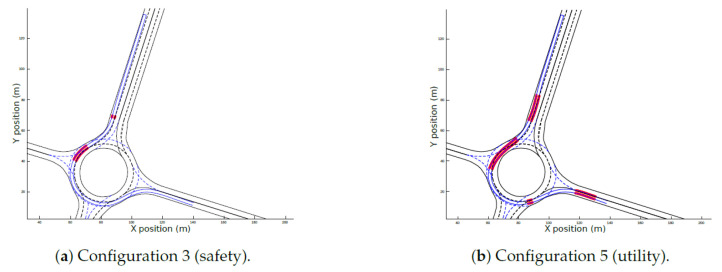
Lane invasion for the simulated environment.

Figure 18a shows the lane invasion for the configuration with best performance in this KPI (Configuration 3), whereas the worst performance, obtained in Configuration 4, is plotted in Figure 18b. The red zones highlight the areas when adjacent lanes were occupied by the ego-vehicle during the travel. In Configuration 3, where safety was prioritized, the lane invasion is low, and even in the red zones, the distance to the border of the lane $\left(d_{li}\left(s\right)\right)$ is almost zero. Conversely, as the priority of Configuration 4 was utility, straighter candidates with higher speeds were chosen, even if it that decision compromised safety by invading adjacent lanes.

**Figure 19:**
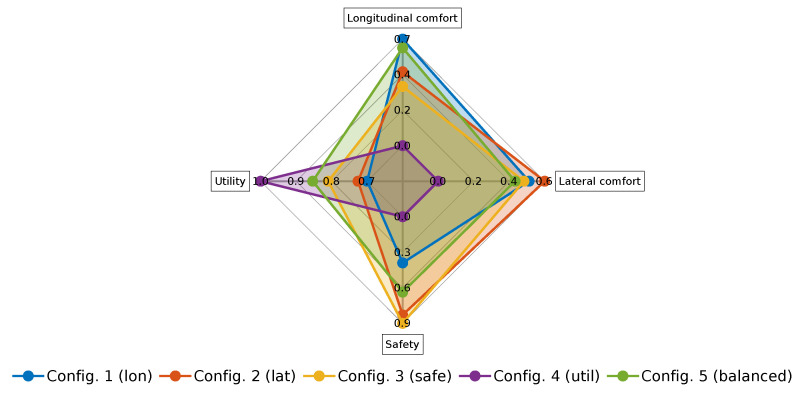
Radar plot of the comparing categories for the different configurations performed in the simulation environment.

Table 6 shows the numeric values of the resulting KPI in these configurations, and Figure 19 shows a radar plot of the comparing categories after combining the KPI. Configuration 1 showed good performance on longitudinal and lateral comfort, but it had medium and poor performance on safety and utility, respectively. Configuration 2 had good performance in longitudinal/lateral comfort and safety, but it also presented a poor performance with respect to utility. Configuration 3 exhibited a good behavior in terms of lateral comfort and safety, and a medium performance on longitudinal comfort and utility. Configuration 4 had the worst performance in all categories except in utility, where it had the best behavior. The balanced configuration showed a medium-high performance in all categories, with longitudinal comfort almost being the highest one.

**Figure 20:**
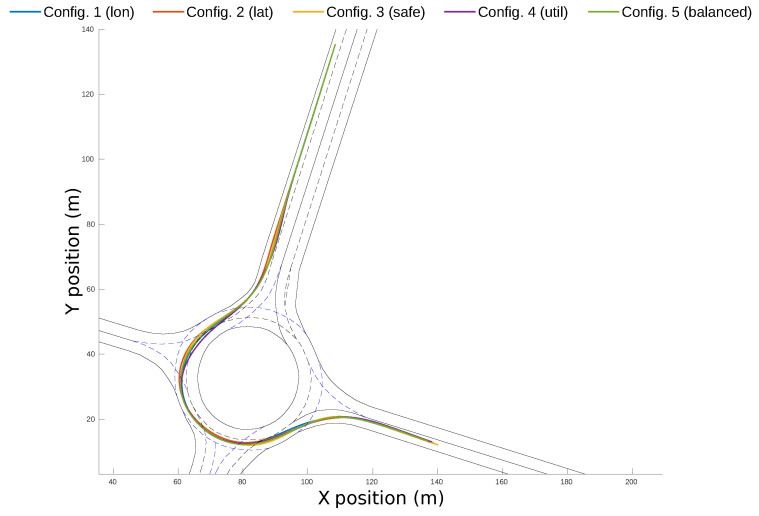
Final trajectories for the different weight configurations in the simulation environment.

Figure 20 shows the complete path of each configuration along the roundabout scenario. Even though the configurations follow similar paths, in Configuration 4, the lane invasion on the initial segment of the roundabout is very prominent. It can also be observed that Configuration 3 is close to the centerline during the whole scenario.

#### 5.2.2. Real Vehicle Experiments

**Figure 21:**
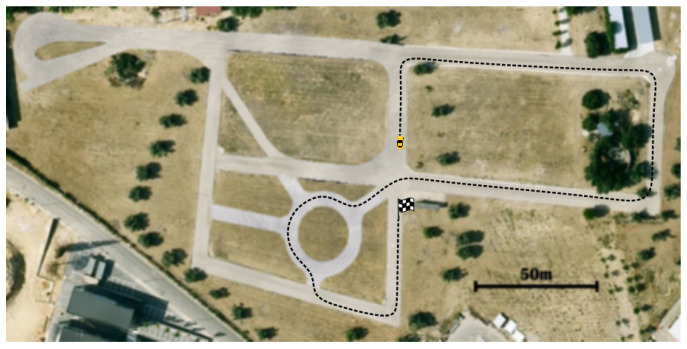
Route to be followed in the experiments with real vehicle.

The trials were carried out in the proving ground of the Centre for Automation and Robotics (CSIC), Spain. The vehicle executed three 90° turns before facing a roundabout, then took the third exit and made one last turn before reaching the destination point. Figure 21 shows an aerial view of the testing facilities and the complete route used in this experiment.

**Figure 22:**
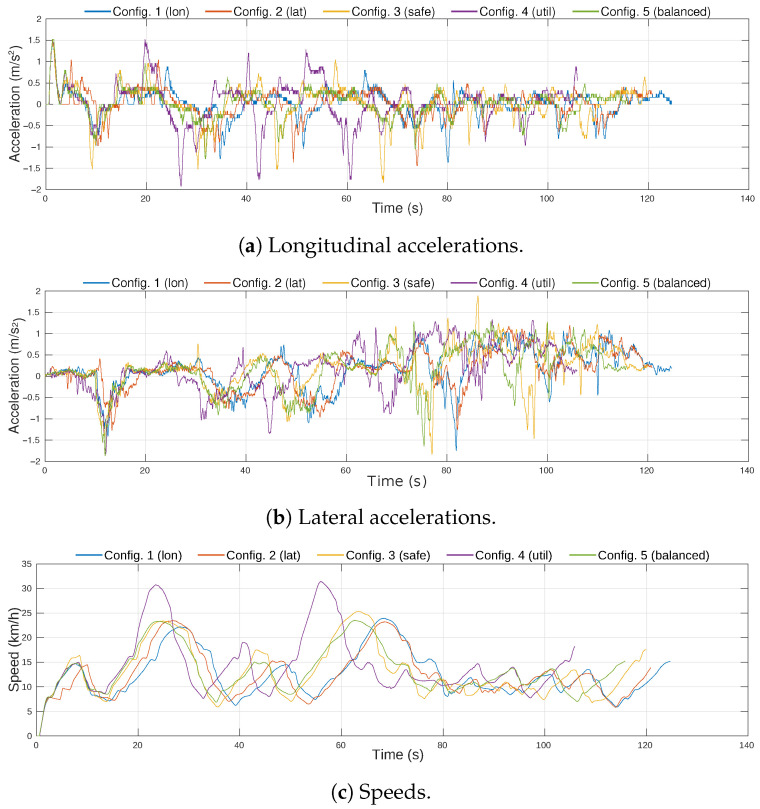
Acceleration and speed profiles for the different configurations executed in the real environment.

Figure 22 shows the acceleration and speed profiles of the five configurations. These data were obtained from the on-board sensors of the vehicle. Since there were no other vehicles involved in this setup, the differences between configurations are higher than in the simulation environment. Regarding longitudinal accelerations (Figure 22a), Configuration 1 and Configuration 5 had the lowest values over time, and Configuration 4 presented the highest magnitudes of both positive and negative accelerations. With respect to lateral accelerations (Figure 22b), Configuration 1 and Configuration 2 kept the lower and more stable values along the route. In the speed profiles graph (Figure 22c), it can be observed that Configuration 4 reaches 30 km/h twice during the route, while the other configurations did not exceed 25 km/h. The traveling time was significantly different in all the configurations, being Configuration 4 the fastest with a traveling time of 105 s, and Configuration 1 the slowest with a traveling time of 124 s.

**Figure 23:**
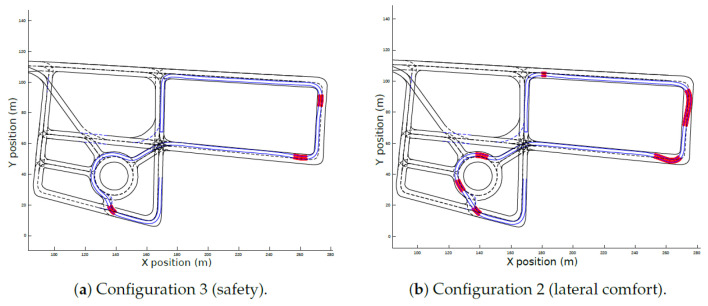
Lane invasion for the real environment.

Once again, Configuration 3 presented the best performance on the lane-invasion KPI (Figure 23a). The worst performance was obtained in Configuration 2 (Figure 23b) because the curves of the test track were very sharp and the planner decided to make wider turns in order to maximize the lateral comfort. In Configuration 3, the magnitudes of $d_{li}\left(s\right)$ were almost zero, while in Configuration 2, the magnitudes of $d_{li}\left(s\right)$ are higher.

**Figure 24:**
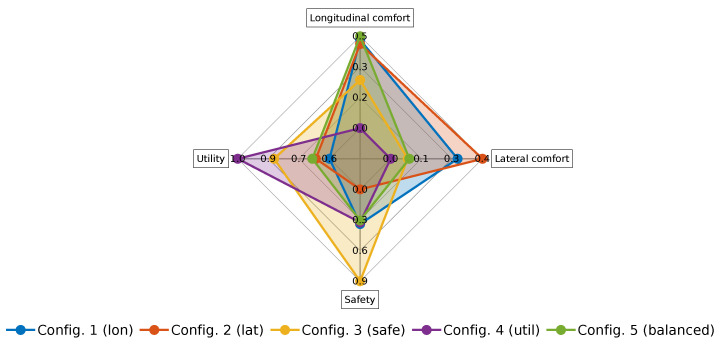
Radar plot of the compared categories in the real vehicle experiments.

Table 7 shows the numeric values of the resulting KPI and Figure 24 shows a radar plot of the comparing categories after combining the KPI. In the case of the real vehicle experiments, Configuration 1 also showed good performance on longitudinal and lateral comfort, at the expense of sacrificing safety and utility. Configuration 2 had very good behavior in terms of longitudinal and lateral comfort, but it had the worst safety performance because, in order to preserve lateral comfort, higher lane-invasions were applied; it also presented a poor performance in terms of utility. Configuration 3 had a medium performance longitudinal/lateral comfort and utility, but it exhibited the best performance by far in terms of safety. Configuration 4 had the worst performance in the longitudinal and lateral comfort categories, but it was reasonably safe and scored the best results in terms of utility. The balanced configuration showed a medium performance in all categories, having again an above average result in longitudinal comfort.

**Figure 25:**
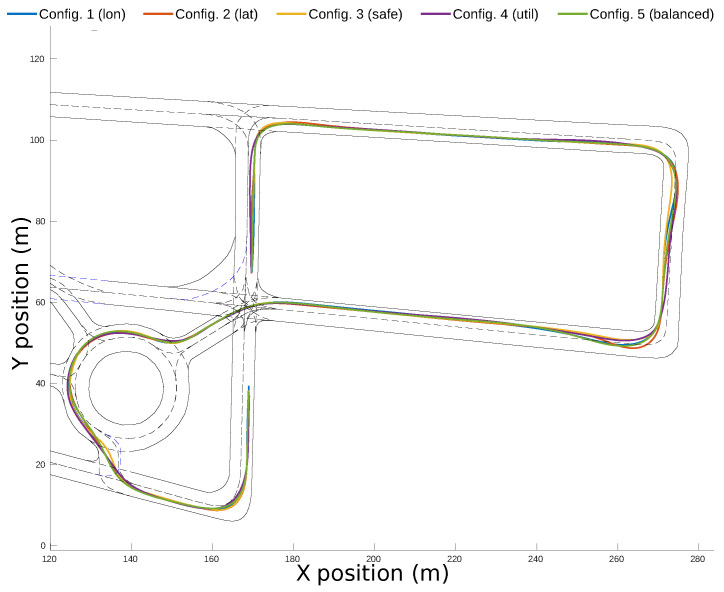
Final trajectories for the different configurations in the real environment.

Figure 25 show the complete path of each configuration along the test-track. It is important to notice how the trajectory followed by the ego-vehicle tends to maximize comfort and speed. Indeed, it can be observed that it is closer to the inner border of the lanes during the first part of the turns and thereafter moves closer to the outer border. Configuration 2 had the highest lane-invasion along the route, and Configuration 3 was closer to the centerline.

#### 5.2.3. Results Comparison

After performing the experiments using five weight configurations in simulated and real environments, there are some relevant aspects that are worth mentioning. In both environments, there was a strong correlation between lateral and longitudinal comfort: if one of these DV was prioritized, the other enhanced its performance accordingly. The lane invasion was reduced drastically in both environments when the safety DV had the highest weight; nevertheless, the worst scenario in the simulated environment was Configuration 4 (utility), while in the real environment it was Configuration 2 (lateral comfort); this disparity is because, in the case of the simulated environment, the lane invasion was produced when straighter (and faster) trajectories were selected inside the roundabout, while in the case of the of the real environment, since the turns are more narrow, the opposite lane had to be invaded in order to make wider and more comfortable turns. In both environments, the utility had a negative impact on the rest of the DV, introducing the dilemma of choosing between comfortable and safe travel vs. a fast and more aggressive maneuver. In the case of the real environment, the utility presented a greater impact on the traveling speed due to the lack of traffic agents that may limit the speed of the ego-vehicle.

Note also that both longitudinal and lateral accelerations reached higher values in the real environment, as a result of the narrow turns of the test track. The average jerk values were also higher in the real environment, which was the reason for this the noisy signals of the accelerometers on board the automated vehicle.

#### 5.2.4. Computing-Time Results

The experiments in simulation were conducted on a computer with an Intel Core i7-7700 3.6 GHz processor and 16 GB RAM, while on the real vehicle, the processing unit was an Intel Core i7-7700HQ 2.8 GHz processor with 16 GB RAM.

**Figure 26:**
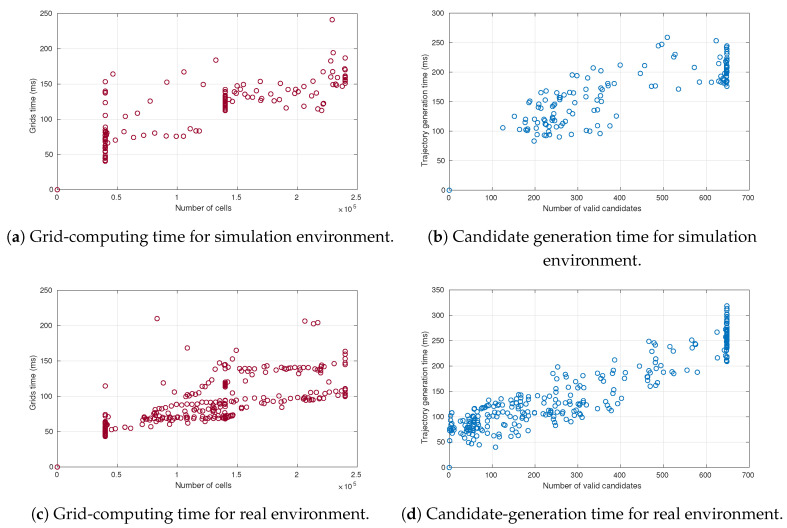
Times for grid and candidate generation.

Figure 26 shows the computing time analysis for the two main stages in the motion-planning algorithm: grid-computing and candidate-generation. Figure 26a,c, shows computing times for the grids at each iteration of the algorithm. It can be seen that it increases with the number of cells. In the case of the simulation environment, the grids’ computing time was higher on average than in the real environment due to the need to generate prediction grids for each vehicle present in the scene. Figure 26b,d, shows the time needed to create the trajectory set. It shows a proportional relation between the number of valid candidates and the computing time for generating them. The point distribution in the figures highlights that the simulation environment allowed to obtain more valid candidates along the route than the real environment (83% versus 60%, respectively). This difference can be explained by the different geometry of the roads, as turns are tighter in the real test track, leading to lower number of valid candidates.

Table 8 shows the average computing times of the motion planning algorithm in simulation and real environments. It can be observed that the average of planning time was around 400 ms for 650 trajectory candidates in both environments.

## 6. Concluding Remarks

This paper presents a merit-based motion planning algorithm for an autonomous driving system, which allows to customizing the driving profile according to four decision variables.

The motion planning strategy was tested by executing a set of experiments on urban-like scenarios with different weight configurations and comparing the final trajectories and relevant KPI. The results showed that when comfort criteria has higher priority, the resulting acceleration profiles are smoother and have lower overall values, both in longitudinal and lateral comfort. When safety is considered the most important design variable, the resulting trajectories do not invade adjacent lanes, avoiding possible collisions with near obstacles. When the priority is efficiency or utility, the generated trajectories are faster and exhibit more aggressive acceleration profiles.

The system was tested using software-in-the-loop in a simulated roundabout scenario with traffic, and it was able to obtain a comfortable and safe trajectory by avoiding the dynamic obstacles present on the scene while keeping bounded the lateral and longitudinal accelerations. The system was also tested successfully in a real vehicle on a test track that includes intersections and a roundabout.

Since the trajectory generation algorithm constantly generates a variety of candidates to the reachable lanes of the ego-vehicle, a fall-back maneuver strategy can be implemented in the future. Indeed, in case the current trajectory is no longer valid, a valid candidate is always available as a backup. A machine-learning algorithm may also be implemented to automatically infer the most adapted weights for the merit-function to match a driving profile.

## Figures and Tables

**Table 1:** Trajectory Performance Indicators and decision variables of trajectory candidates.

Decision Variable	*TPI*	Formula
Longitudinal comfort	Average acceleration	$\bar{|\gamma_{x}\left(s\right)|}$
	Maximum acceleration	$max(\gamma_{x}{\left(s\right)}^{2})$
	Average jerk	$\bar{|{ȷ}_{x}\left(s\right)|}$
	Maximum jerk	$max({\;{ȷ}_{x}\left(s\right)}^{2})$
Lateral comfort	Average acceleration	$\bar{|\gamma_{y}\left(s\right)|}$
	Maximum acceleration	$max(\gamma_{y}{\left(s\right)}^{2})$
	Average jerk	$\bar{|{ȷ}_{y}\left(s\right)|}$
	Maximum jerk	$max({\;{ȷ}_{y}\left(s\right)}^{2})$
	Smoothness	$\int_{s_{0}}^{s_{f}}{\dot{k}\left(s\right)}^{2}+w_{\ddot{k}}*{\ddot{k}\left(s\right)}^{2}ds$
Safety	Safe chase	$\bar{\hat{d}\left(s\right)/d_{0}}$
	Closeness	$max(G_{occ}^{i})$
	Occupancy	$\bar{G_{occ}^{i}}$
	Lane invasion	$\bar{G_{inv}^{i}}$
Utility	Path length	$L_{max}-L$
	Average speed	$v_{max}-\bar{v(s)}$

**Table 2:** Weight configurations of decision variables for use case scenario.

Decision Variable	Config. 1	Config. 2	Config. 3
Longitudinal comfort	0.5	1.0	0.1
Lateral comfort	0.5	0.1	0.1
Safety	0.5	0.1	0.1
Utility	0.5	0.1	1.0

**Table 3:** Weight configurations of the decision variables for different experiments.

Decision Variable	Config. 1	Config. 2	Config. 3	Config. 4	Config. 5
Longitudinal comfort	1.0	0.1	0.1	0.1	0.8
Lateral comfort	0.1	1.0	0.1	0.1	0.8
Safety	0.1	0.1	1.0	0.1	0.8
Utility	0.1	0.1	0.1	1.0	0.8

**Table 4:** Acceleration limits used in the validation experiments.

Parameter	Unit	Value
$\gamma_{comf,lat}$	(m/s${}^{2}$)	1.0
$\gamma_{comf,acc}$	(m/s${}^{2}$)	1.1
$\gamma_{comf,dec}$	(m/s${}^{2}$)	1.2
$\gamma_{safe,dec}$	(m/s${}^{2}$)	4.0

**Table 5:** Key performance indicators used to compare the performance of different configurations.

Comparing Category	*KPI*	Formula
Longitudinal comfort	Average acceleration	$\bar{\gamma_{x}{\left(s\right)}^{2}}$
	Average jerk	$\bar{{ȷ}_{x}{\left(s\right)}^{2}}$
Lateral comfort	Average acceleration	$\bar{\gamma_{y}{\left(s\right)}^{2}}$
	Average jerk	$\bar{{ȷ}_{y}{\left(s\right)}^{2}}$
Safety	Lane invasion	$\int_{s_{0}}^{s_{f}}d_{li}\left(s\right)\;ds$
Utility	Positive acceleration	$\int_{s_{0}}^{s_{f}}max(\gamma_{x}\left(s\right),0)\;ds$
	Average speed	$\bar{v_{s}}$

**Table 6:** Key performance indicators obtained in the simulation environment.

Decision Variable	*KPI*	Config. 1	Config. 2	Config. 3	Config. 4	Config. 5
Longitudinal comfort	Average acceleration (m/s${}^{2})$	0.091	0.143	0.174	0.268	0.123
	Average jerk (m/s${}^{3})$	0.957	1.621	1.834	3.030	0.943
Lateral comfort	Average acceleration (m/s${}^{2})$	0.113	0.115	0.119	0.143	0.129
	Average jerk (m/s${}^{3})$	0.018	0.012	0.018	0.055	0.020
Safety	Lane invasion (m)	109.591	30.522	17.796	179.785	65.188
Utility	Positive acceleration (m/s${}^{2})$	139.932	153.225	200.556	280.480	212.376
	Average speed (m/s)	12.502	12.547	12.463	13.951	13.209

**Table 7:** Key performance indicators to compare the performance of different configurations in the real environment.

Decision Variable	*KPI*	Config. 1	Config. 2	Config. 3	Config. 4	Config. 5
Longitudinal comfort	Average acceleration (m/s${}^{2})$	0.101	0.108	0.169	0.250	0.100
	Average jerk (m/s${}^{3})$	1.732	1.761	1.965	2.246	1.650
Lateral comfort	Average acceleration (m/s${}^{2})$	0.226	0.221	0.262	0.298	0.287
	Average jerk (m/s${}^{3})$	0.844	0.612	1.203	1.233	1.091
Safety	Lane invasion (m)	264.25	405.51	31.45	270.92	279.01
Utility	Positive acceleration (m/s${}^{2})$	177.200	215.440	325.200	396.880	208.560
	Average speed (m/s)	12.403	12.428	12.862	14.407	13.271

**Table 8:** Computing-time analysis for the experiments performed in simulation and real environments.

Environment	Grids Time	Candidates Generation	Remaining Tasks	Total Time
Simulation Environment	115.7±40.3 ms	165.3±48.8 ms	137.6±58.4 ms	418.6±79.9 ms
Real Environment	89.3±33.7 ms	150.4±71.9 ms	157.4±57.1 ms	397.1±82.2 ms

## Data Availability

Not applicable.
